# Supportive Care Needs of Young Adults With Endometriosis: An Open‐Ended Online Survey and Exploration of Unmet Needs

**DOI:** 10.1111/hex.70045

**Published:** 2024-10-02

**Authors:** Louis Taffs, Niamh Waters, Jennifer Marino, Charlene Rapsey, Michelle Peate, Jane E. Girling

**Affiliations:** ^1^ Department of Obstetrics, Gynaecology, and Newborn Health The Royal Women's Hospital and The University of Melbourne Melbourne Australia; ^2^ Faculty of Medicine and Health, Sydney Medical School The University of Sydney Sydney Australia; ^3^ Department of Psychological Medicine, Dunedin School of Medicine University of Otago Dunedin, Aotearoa New Zealand; ^4^ Department of Anatomy, School of Biomedical Sciences University of Otago Dunedin, Aotearoa New Zealand

**Keywords:** adolescent, dysmenorrhoea, dyspareunia, endometriosis, pelvic pain, quality of life, unmet needs

## Abstract

**Objective:**

The aim of this study is to identify and explore the unmet needs of adolescents and young adults living with endometriosis.

**Design:**

An open‐ended online survey was conducted, with questions derived from prior research looking at areas of unmet need in healthcare, career and work, financial, information, psychological, social and cultural domains.

**Setting and Population:**

Self‐selecting 18‐25 year olds with surgically diagnosed endometriosis (self‐reported) currently living in Australia were included as participants.

**Methods:**

Invitation to participate in an open‐ended online survey was shared through the social media of Australian endometriosis organisations and the Royal Women's Hospital, Melbourne. Surveys were analysed qualitatively through template analysis.

**Main Outcome Measures:**

Recording of the unmet supportive care needs of this population was carried out.

**Results:**

One hundred and thirty‐one respondents fit the eligibility criteria of being aged 18–25 years (median age 23 years). Most were born in Australia (94%), university‐educated (54%) and lived in a metropolitan setting (69%). There was a range of unmet needs that were presented across education, work, healthcare and relationships. Group‐specific challenges were identified: doctors either over‐ or underemphasising future fertility; disrupted sexual and romantic life due to painful sex; managing pain in the classroom and workplace where periods are taboo; and being gender‐queer in gynaecological medical spaces.

**Conclusions:**

The increasingly young age at which patients are receiving an endometriosis diagnosis precipitates a shift in patient care. The treatment decisions that are being made must be reflective of the unique needs of the adolescents who carry the burden of the disease. Clinicians are advised to be aware of and discuss needs with their patients.

**Patient or Public Contribution:**

The nine open‐ended questions in this survey were developed from data from a preliminary series of interviews with endometriosis patients in a tertiary women's healthcare centre. In asking these data‐informed questions to the online endometriosis community, patients across broader sociocultural demographics and disease states (including less symptomatic endometriosis) have provided a broader understanding of their supportive care needs.

## 1 Introduction

Endometriosis is a gynaecological disease that affects 600,000 to 700,000 women and people presumed female at birth in Australia and 200 million globally [1]. It is defined by the presence of endometrial‐like tissue outside of the uterus [2]. This disease has an unknown aetiology and a range of symptomatic presentations [3]. Most commonly, endometriosis causes painful menstruation, heavy bleeding, pain during or after sexual activity, and infertility [2, 4]. The social and financial impacts of this condition are being increasingly recognised in Australia [5], with the federal government creating a national plan for endometriosis and allocating funding to allow for greater public access to gynaecological services [6, 7].

One strategy to improve the day‐to‐day lives of endometriosis patients is the development of a survey instrument to identify an individual's supportive care needs [8]. The supportive care needs framework recognises that an individual's unmet needs are the translation from the experience of their symptoms to their self‐reported Quality of Life (QoL) [9, 10]. A patient with severe symptoms, but whose many needs are met, will report a higher QoL than a patient with more mild symptoms, but with needs unidentified or neglected [11]. Supportive care needs survey instruments aim to identify these needs, allowing healthcare professionals, patients and families to work together to address them and enhance patient well‐being [8]. Although there is increasing literature on the impact of endometriosis [12], research specifically asking these patients their needs, and engaging patient demographics whose experience have been less explored, is necessary to construct such an instrument [8]. Although there is information on the impacts of endometriosis on adolescents and young adults (AYA) [13, 14, 15, 16, 17] and the unmet needs of adolescents with menstrual symptoms are been explored [18, 19], there is little research specifically on the unmet supportive care needs of the AYA population with endometriosis.

Historically, endometriosis has not been considered a disease of young people [20, 21]. However, as public awareness of endometriosis increases and as less invasive forms of diagnosis are becoming increasingly accepted [22, 23], the diagnostic delay of endometriosis is decreasing (in Australia and Aotearoa New Zealand) [12, 24]. This reduction of diagnostic delay, as well as recognition of the impact of uncontrolled disease in adolescence [25], is resulting in the initiation of endometriosis management at an increasingly younger age.

Both the disease and their experience of it can be different for young adults [26]: a combination of symptomatic differences, life goals and supportive care needs [18]. For example, young people report endometriosis‐related dyspareunia more often than their older counterparts [13, 14, 27]. The challenges in managing and communicating about painful sex are likely to be different for a married 40‐year‐old compared with a 20‐year‐old in a time of rapid formation of identity and relationships [27, 28].

When an AYA is diagnosed, they are burdened with the consequences of complex treatment decisions for their lifetime [26]. Therefore, an understanding of the adolescent endometriosis patient's current needs, as well as an appreciation for their needs in the future, is paramount. To this end, we aimed to collate and understand the needs, met and unmet, of AYA patients living with endometriosis [8, 11].

## 2 Methods

### 2.1 Study Design

A qualitative cross‐sectional survey was conducted.

### 2.2 Ethics

The design of the survey and the collection of the data were approved by the University of Melbourne Human Research and Ethics Committee (HREC) 2056522. All participants provided informed electronic consent.

### 2.3 Measures

Nine open‐ended questions were derived from earlier qualitative research with endometriosis patients attending The Royal Women's Hospital, Melbourne, for endometriosis treatment [29]. The 12 participants of these interviews had an average age of 30 years (range 23–42). These interviews ensured that the nine topics asked in this anonymous online survey had a basis in the lived experience of endometriosis patients. However, they were not specific to the AYA population. The questions were worded to encourage a needs‐based framing of participant thinking and answers: ‘Can you think of any needs or wants that relate to…’ doctors or other medical practitioners; healthcare or access to care in general; relationships and social life; daily living; access to information; financial matters; employment or education; emotions, spirituality and culture and other.

The survey also collected demographic information and a brief history of their endometriosis symptom load and diagnostic timeline (details are presented in Tables 1 and 2).

**Table 1:** Demographic Information.

	*n* (%)
Age	
18	4 (3.1)
19	7 (5.3)
20	10 (7.6)
21	17 (13.0)
22	24 (18.3)
23	21 (16.0)
24	24 (18.3)
25	24 (18.3)
Sex assigned at birth	
Female	131 (100.0)
Male	0 (0.0)
Identify as	
Female	130 (99.3)
Gender‐non–conforming or non‐binary	1 (0.8)
Male	0 (0.0)
Ethnicity^a^	
Anglo‐Saxon/English	114 (87.0)
Italian	6 (4.6)
Aboriginal/Torres Strait Islander	4 (3.1)
Greek	4 (3.1)
Indian	3 (2.3)
Maltese	2 (1.5)
Croatian	2 (1.5)
Other^b^	7 (5.3)
Born in Australia	
Yes	123 (93.9)
No	8 (6.1)
Highest level of education	
University	73 (55.7)
TAFE/College	28 (21.4)
Highschool	30 (22.9)
Rurality	
Major cities	90 (68.7)
Inner regional	28 (21.4)
Outer regional	8 (6.1)
Remote	2 (1.5)
Very remote	3 (2.3)
State	
Victoria	64 (48.9)
New South Wales	27 (20.6)
South Australia	13 (9.9)
Western Australia	12 (9.2)
Queensland	11 (8.4)
Australian Capital Territory	3 (2.3)
Northern Territory	1 (0.8)
Tasmania	0 (0.0)

^a^ Totals sum to greater than 100%, as participants could choose multiple ethnicities.

^b^ Other categories included ethnicities Lebanese, Latin American, Brazilian, Anglo‐Asian, Jewish, Irish and Serbian (*n* = 1 for each).

**Table 2:** Endometriosis history.

	*n* (%)
Symptoms	
Pelvic pain around the time of your period	130 (99.3)
Pelvic pain not at the time of your period	123 (93.9)
Infertility	14 (10.7)
Heavy bleeding	107 (81.7)
Pain during or after sexual activity	102 (77.9)
Pain during or after using your bowels	105 (80.2)
Pain during or after using your bladder	85 (64.9)
Other symptoms, including bloating, nausea, migraines, non‐pelvic pain, gastro‐intestinal disturbances and fatigue.	59 (45.0)
Years since diagnosis	
Less than a year	35 (26.7)
1–5 years ago	82 (62.6)
5–10 years ago	13 (1.0)
Over 10 years ago	1 (0.1)
Years since onset of symptoms	
Less than a year	0 (0.0)
1–5 years ago	27 (20.6)
5–10 years ago	68 (51.9)
Over 10 years ago	36 (27.5)

### 2.4 Recruitment

Participants were invited to complete the survey through social media posts distributed via Australian women's health organisations and endometriosis support networks, namely, The Royal Women's Hospital, Endometriosis Australia, EndoActive and Endogram. The posts are linked to a study information page. All participants provided informed electronic consent before being directed to the survey.

Data were collected and managed using Research Electronic Data Capture (REDCap) [30], a secure web‐based software platform designed to support data capture for research studies, hosted at The University of Melbourne.

### 2.5 Eligibility

Eligibility criteria included anyone living in Australia, over the age of 18 years, who had received a diagnosis of endometriosis from a medical professional (self‐reported). From this cohort, to capture the needs of AYA, responses from participants aged between 18 and 25 years (inclusive) were then extracted for the current analysis. Data were collected during the dates of 24 May 2020–14 November 2020. Access to a computer or smart device was necessary to complete the survey.

### 2.6 Analysis

Survey data were managed and analysed in NVivo (QSR International v.12) [31]. Utilising a template analysis framework, the data were analysed for content themes, independently double‐coded by researchers L.T. and N.W. with > 95% initial congruency [32]. Discrepancies were settled by consensus. This analysis was focused on extracting what could be qualified as ‘needs’ within a supportive care needs framework (i.e., organised under the domains of Healthcare, Career and Work, Financial Impact, Information, Psychological and Emotional and Social Cultural identified in prior work) [29]. These initial domains were then re‐examined by the author and consolidated into four care domains of need. Although interpretation of participants’ writing is inevitable, as some participants implied needs rather than explicitly writing ‘I need…’. The needs presented in Table 3 are largely descriptive.

**Table 3:** Domains, themes and needs. Needs identified by participants have been summarised in the form of a statement, beginning with 'I need…'. ‘*n*’: the number of participants who mentioned the need (from a total of 131).

Domain	Theme	Need (number of respondents)
1.0 Health *n* = 128	1.1 Access	I need my healthcare to not be financially burdensome. (*n* = 90)
		I need shorter wait times for the public system. (*n* = 16*)*
		I need more specialists for endometriosis. (*n* = 13)
		I need endometriosis surgeries be classified as nonelective. (*n* = 8)
		I need local access to endometriosis care. (*n* = 4)
		I need a centralised centre for my all my endometriosis care needs. (*n* = 4)
	1.2 Quality of Care	I need my health practitioners to be better informed about endometriosis. (*n* = 58)
		I need allied health, psychology and holistic health practices to be actively included in my healthcare. (*n* = 46)
		I need my doctor to provide me with information about endometriosis and to make a management plan for the future. (*n* = 45)
		I need more effective treatment, with fewer side effects. (*n* = 41)
		I need faster diagnosis and appropriate referrals. (*n* = 33)
		I need more good research to be done on endometriosis. (*n* = 23)
	1.3 Quality of Interaction	I need to be understood and listened to by health practitioners. (*n* = 60)
		I need to lead the decisions regarding my health. (*n* = 11)
		I need endometriosis to be considered as a diagnosis, even if I am young. (*n* = 11)
		I need to know about all options for my future fertility at diagnosis, regardless of age or life stage. (*n* = 7)
		I need healthcare practitioners to be familiar and comfortable with trans, nonbinary and gender diverse peoples. (*n* = 2)
2.0 Home *n* = 66	2.1 Daily Symptom Management	I need help with my sex life. (*n* = 21)
		I need access or education regarding non‐pharmaceutical pain relief, like hot water bottles, TENS machines, or exercise. (*n* = 19)
		I need to be able to follow a diet that helps with my endometriosis and know what that diet is for me. (*n* = 6)
		I need access to legal cannabis. (*n* = 6)
		I need products that make my life easier. (*n* = 2)
	2.2 Support at Home	I need support and education available for the people that support me. (*n* = 29)
		I need to be understood and listened to by my partner and/or my family. (*n* = 14)
		I need assistance with daily activities at home. (*n* = 4)
3.0 Work *n* = 103	3.1 Support at Work/School	I need my employer/educator to be better informed about endometriosis. (*n* = 55).
		I need my employer/educator to be more flexible with workload and deadlines. (*n* = 44)
		I need my employer/educator to not discriminate against me because of my endometriosis. (*n* = 30)
		I need more sick leave available to me. (*n* = 28)
		I need my place of work/education to have appropriate facilities for me. (*n* = 8)
	3.2 Systematic Support	I need the government to class endometriosis as a disability. (*n* = 36)
		I need to find employment that does not make my endometriosis worse. (*n* = 4)
4.0 Society *n* = 105	4.1 Understanding and Awareness	I need public education, awareness and understanding. (*n* = *6*4)
		I need public education initiatives for endometriosis and other gynaecological conditions to be talked about in high schools. (*n* = 32)
		I need a centralised access point for information that is up to date, accurate, and consumable. (*n* = 31)
		I need there to be less stigma and taboo around periods. (*n* = 5)
		I need access to support groups, and to talk to other people with endometriosis. (*n* = 9)
	4.2 Facilities	I need more public facilities and to be able to access them. (*n* = 3)

Due to the large number of responses, and because there is no way to refine and ask further questions in an online survey, the number of participants who related each need is reported in Table 3. However, there was no minimum threshold number of mentions of a theme to be included in reporting, and all needs were given equal weighting in the generation of themes, even if they were present in only a single participant's response. The decision to include all needs was to try to ensure that no person was overlooked, or minimised, due to our interpretation.

Illustrative participant quotations are presented under pseudonyms. Rurality was determined using the *Modified Monash Model* [33].

### 2.7 Reflexivity Statement

The primary author of this article is a young male medical student, who, at the time of data collection analysis, fell into the age bracket of AYA that is examined here. When I began this study, I had one friend with whom I had talked about their endometriosis diagnosis. By the time of writing this manuscript and since it had become known that I was working on this study, I have had many more friends, acquaintances, peers and sometimes strangers tell me their stories. Although their stories are not included as data points or in any formal analysis, these conversations would have impressed upon me the importance of this topic and some of the issues facing AYAs trying to manage their endometriosis.

## 3 Results

### 3.1 Participant Demographics

Of 512 completed surveys, 131 (25.6%) were from people aged 18–25 years, and thus eligible for this analysis. Respondents within this range had a median age of 23 years, were mostly university‐educated (55.7%), predominantly of Anglo‐Saxon/English descent (87.0%), lived in a metropolitan area (68.7%) and were from the state of Victoria (48.9%) (Table 1).

Dysmenorrhoea (99.3%) and non‐cyclic pelvic pain (93.9%) were the two most frequently recorded symptoms, followed by menorrhagia (81.7%), dyschezia (80.2%) and dyspareunia (77.9%) (Table 2). Most respondents had been experiencing symptoms for more than 5 years (79.4%).

### 3.2 Needs

In general, there were two styles of responses. The first explicitly addressed needs with short statements (e.g., I need…). The second provided context for their situation and frequently offered potential solutions to the problems that they were facing. Some participants asked for a problem to be solved, whereas others asked for tools or resources to solve it themselves. Response length ranged from 4 to 1664 words (median 169 words). This analysis focused on both directly provided and implied needs in participant responses.

We identified 37 distinct needs (Table 3), which were grouped under four care domains: Health, Home, Work and Society. In section 3 of this article, we highlight some overarching themes present in these 37 needs. Some themes can be found in multiple care domains, and with relevant domains given in parentheses.

### 3.3 Challenges in Navigating and Acquiring Healthcare as a Young Person (Health)

The healthcare spaces that these young people navigated included allied health, general practitioners, specialists and, for some, the emergency department. Respondents were often well versed in the nuances of private and public healthcare systems in Australia.

Australia has a predominantly public healthcare system with reduced cost to the patient. However, the financial burden of healthcare for managing endometriosis was commonly described as an area of need for young people. Reported expenses included costs of tests and scans from private operators, medications and specialist appointments. Specialist appointments need to be paid by the patient only if they attend privately, but in the public system, participants reported long wait times. Therefore, sometimes, participants felt that the decision of whether to attend privately was out of their hands, as even though they could not necessarily afford to pay for a private specialist, neither could they afford illness‐related work absences and potential job loss while waiting up to 18 months for a public appointment.

It's part of a vicious cycle, too ‐ women with endo often miss work or can struggle to find long term employment, making it even harder to afford specialist medical treatment and medications.

Jessica, Age 22

Many wanted improved quality of medical care: respondents felt that doctors needed to be educated on the benefit of holistic health practices and the necessity of psychological support. It was suggested that information about and referrals to these services should be provided by doctors when an initial diagnosis of endometriosis is suspected or formally given. Participants expected that these services should collaborate to provide the best care for the patient. Some respondents stated that they needed access to psychologists or counsellors who specialise in endometriosis or other chronic pain diseases.

The respondents in this survey reported facing difficulties in accessing endometriosis care because of their youth. They reported that healthcare professionals determined that their pain was a result of their adolescent bodies adjusting to menstruation and would subside with time. This caused a loss of trust in professionals when the pain remained. The real and lasting consequence of this lack of medical care was both the loss of trust in the profession and the feeling of a lost opportunity to begin treatment earlier or make life decisions with the appropriate knowledge of their condition. Some respondents reported that they needed more care or counselling regarding their fertility‐related symptoms.

I needed to access information and education when I was first diagnosed (as a teenager) regarding what measures (lifestyle or medical) I should be doing and considering in order to best protect my chances of fertility and that would limit my need for future surgeries. I did not receive this information, I was told instead that I was too young to be concerned with those issues, despite me already being concerned with those issues.

Kate, Age 21

Others reported that they felt that too much consultation time was spent on their fertility, even when it was something that they were not concerned about. Some respondents felt that some treatment options (such as hysterectomy) were not explored because of their impact on future fertility.

A lack of inclusive, diverse language was a problem. Two respondents reported that some doctors were not culturally aware of queer people.

I want doctors to believe me and other AFAB people who have pain… I want doctors and gynaecologists to be familiar and comfortable with patients with endometriosis who are non‐binary, trans, and gender diverse who don't identify as women.

Alex, Age 25

#### 3.3.1 Need for Non‐Medical Management of Pain (Health, Home, Work)

Respondents reported a variety of needs relating to self‐management of pain at home or work. Respondents needed information on techniques, or access to products like TENS machines and electric heat packs, to manage pain. For some, learning about diet was important, as well as research discerning which diet was best suited for people with endometriosis. Cannabis products were a self‐management strategy of interest to or used by some, but barriers included legality and cost of legal access. These people needed easier medical access to pain relief that worked for them.

The importance of physical activity in reducing pain was highlighted by some, particularly in its absence due to COVID‐19 restrictions at the time of data collection. For others, COVID‐19 led them to realise that the mobility required of them in normal, non‐pandemic life was a factor in their pain. These people needed flexible work‐from‐home arrangements to facilitate days where mobility was limited. Some people needed work‐from‐home arrangements because non‐medical pain relief options, like baths, heat packs and comfortable seating, were readily available.

Mine's been much better during covid because I have been able to conduct my normal life but from home which has made everything more accessible.

Rachel, Age 18

#### 3.3.2 A Supportive Environment (Home, Society)

Respondents included those living with parents, those living out of home and those living with a partner and starting their own family. Regardless of living situation, a supportive and understanding home environment was an area of great need for many respondents. The major barrier to care from partners and family was education about endometriosis and its symptoms. For one respondent, a family history of endometriosis meant that, within the home, its impacts were recognised, and therefore, the home was a caring environment. Another respondent stated that their partner needed education about the realities of the disease, but after receiving this education, he was better equipped to meet her supportive care needs.

I needed my partner to better understand endo and the pain I would sometimes suffer, and after talking to him about it, he listened and learned.

Nicole, Age 25

However, some found educating their partners, family or friends a burden. Respondents wished that they had access to resources that they could share with their loved ones or access to publicly available, promoted education materials.

I think it would be good to have basic information in outsider language that provides them enough information on what it is and what role they play and how they can help. Saves me lots of time and explaining.

Hannah, Age 24

The need for a supportive home environment was highlighted by those who continued to live with their parents into young adulthood. These respondents could not risk moving away from their support systems. Furthermore, some of these respondents identified that their families also needed their own, separate support in the form of information, psychological services or hiring in‐house support workers to aid with their care.

Partners and carers also need to be able to access support for mental health concerns. They may not be able to deal with their emotions, exhaustion and frustration which comes from supporting and seeing their loved ones suffer.

Kate, Age 21

### 3.4 Impact on Relationships and Sex, a Key Issue (Health, Home)

Over three quarters of respondents (77.9%) reported ‘Pain during or after sex’ (Table 2). Respondents expressed a need for information from a sex therapist or doctor regarding managing painful sex, as well as understanding and support from male partners around sex. This tied in with unmet needs regarding partner understanding and support, and educational resources.

In relationships, it can be really hard to describe pelvic pain, painful sex or vaginismus if your partner (if that partner is a cis male) has never experienced these things.

Olivia, Age 25

Some participants framed their endometriosis and painful sex as the barrier to relationships that they felt they needed. This was tied with respondents’ desire for ‘normalcy’, participating in the same relationship milestones as their peers, without fear of pain or embarrassment.

I want to have a relationship even though it's almost impossible for someone at my age with my condition.

Charlotte, Age 21

### 3.5 Institutional Support in Managing Endometriosis in the Workplace or School (Work)

The unmet need in this domain was largely the perceived lack of awareness of, or discomfort in discussing, needs with people in positions of power. For some, this was important because they believed that it would lead to more flexibility from employers or educators, a need for many. There was a need for facilities at work, school or university to help manage symptoms with techniques mentioned in an earlier theme, **‘**Need for non‐medical management of pain’. Examples given were a space to rest, special desk chairs, analgesics and permission to use heat packs. One woman reported that lack of these facilities often resulted in exacerbated pain.

I found on many occasions that enduring the trip home [from work] without earlier access to care meant that the pain intensified beyond a manageable point; had I been able to access a quiet room to lie down, take medication and use a heat pack may have helped mitigate the severity of the pain in these situations.

Jessica, Age 22

When symptoms were severe, participants could not work at home or the office. Many reported the need for more paid sick days.

10 sick days a year for a women diagnosed with endometriosis working full time is not enough paid sick leave to ensure she can have needed surgery during the year and also take off work when the pain is unbearable.

Sophie, Age 25

### 3.6 Educating the Public (Society)

Some respondents reported fear of, or actual, discrimination because of endometriosis. Increased education and awareness, particularly in recognition of endometriosis as a chronic pain condition or disability, was an area of need suggested to reduce discrimination.

Many respondents felt that school was the ideal place to educate the population about menstruation and increase awareness about dysmenorrhoea and heavy menstrual bleeding. Some respondents felt that recognising that their periods were exceptionally painful or heavy would have led to advocating for their health earlier, resulting in earlier access to treatment and timelier management.

For some, the important outcome of increased public knowledge of endometriosis was understanding and support from friends and the wider public. There was anxiety around cancelling plans due to unexpected pain or fatigue.

I also wish I could comfortably say to someone ‘my chronic condition illness is flaring up. I can't get coffee/meet you/I'll have to reschedule’ without having to give someone a lesson about it, I don't want to educate while suffering.

Isabella, Age 21

Respondents had some information needs met by the internet, via support groups and pages on Facebook and Instagram. They were grateful for this support and the advice about how to manage their condition.

However, others stated that online support groups did not meet their information needs, as they found that advice was sometimes conflicting, unclear or outdated. One solution proposed was an online resource that provides up‐to‐date, easily understood and medically accurate information for people with a diagnosis and the people in their lives.

## 4 Discussion

This study documents and categorises the supportive care needs of young adults with endometriosis.

The utility of these data is twofold. First, it outlines some of the practices and services in healthcare that young patients currently find inadequate. This can be used to prompt further research into areas of unmet need and be considered when updating current medical education and practice. Second, as improvements in medical and surgical options to treat endometriosis remain uncertain [34], understanding the current lives and persistent needs of these young patients becomes a priority. These data will aid clinicians in understanding the broader needs of this patient demographic, so that together, clinicians and young patients can better approach complex treatment decisions that best suit a patient's current *and* future needs.

The broad areas of need reported by these young people are congruent with those reported or inferred in qualitative studies of endometriosis in older adults, both regarding their experiences with healthcare professionals [35] and experience of the challenges of daily life with the disease [18, 29, 36, 37, 38, 39]. However, some areas of need are distinct to this patient demographic and require attention from clinicians, administrators and carers alike.

### 4.1 Notable Findings and Interpretations

#### 4.1.1 Sexual Life and Endometriosis

Endometriosis can be a cause of painful sex, a routinely reported, but underappreciated, source of unmet need for endometriosis patients [40]. For the adult population, qualitative explorations have linked dyspareunia with struggles in intimate relationships, impacted psychological health and poorer self‐esteem [36]. Management of and conversation about this symptom are therefore particularly pertinent for adolescents who are navigating a developing sexuality and forming their first romantic relationships [13, 28]. Pressures and taboos towards and surrounding sex for adolescents and young people are complex, particularly when combined with pain during or after sex. This led respondents in our study to ‘opt out’ of dating, as they felt they could not have, or did not deserve, a relationship if it could not include penetrative sex. There are surgical [41], pharmaceutical [42], therapy‐ and education‐based interventions [43] available for dyspareunia that can increase the QoL for endometriosis patients. Young patients should not be excluded from information and treatment options for this important symptom because of their age.

#### 4.1.2 Addressing Fertility, but not Just Fertility

The needs articulated and implied by respondents in this survey indicate complexity in the management of fertility and endometriosis in young people. Part of this complexity arises from the fact that infertility is a common symptom of the disease [44], and that pregnancy was, and still is at times, regarded incorrectly by clinicians as a cure [45]. Endometriosis patients have reported being encouraged by doctors to have children before it is ‘too late’, as well as focusing on young female patients’ future fertility, even when it was of little concern to the patient [46]. This is an issue, as some of the treatment decisions that potentially relieve the pain symptoms of endometriosis, such as hysterectomy and complete or partial oophorectomy, come at the cost of future fertility [44, 47]. Patients in our study felt that they were ‘not allowed’ by their clinician to make the treatment decisions that best fit their values and vision of their future, because their future fertility was valued more highly than their current, daily life, pain‐free.

Conversely, there were also participants in this study who reported that *because* of their youth, their concerns about future fertility were dismissed by their doctors. This caused them distress, as they felt that they would have made different life or treatment decisions had they understood the impact of endometriosis on fertility better. Provision of information and good communication (using a patient‐centred approach) between doctors and patients about fertility has been reported to ease the burden of endometriosis for patients [48].

#### 4.1.3 Endometriosis Care for Gender‐Diverse Patients

This survey had only one respondent who identified as non‐cisgendered and only two respondents who explicitly mentioned the experience of being treated for a gynaecological issue as a non‐woman. However, for those who did mention this need, they did so with an emphasis that warranted exploring here.

With increasing societal acceptance, young people are now more likely to identify as gender‐diverse [49]. Accessing healthcare as a gender‐diverse person can be difficult, and many find the experience distressing [49, 50, 51]. As endometriosis is a gynaecological disease, much of the information and research surrounding it are aimed solely at women, but this can be alienating for those who do not identify as such [48, 51]. It is important that gender‐diverse people feel safe and comfortable in their gynaecological care, not only as a minimum standard of care but also so that patients and clinicians can navigate treatments that impact both endometriosis and pathways of gender affirmation together [50, 52] For example, for trans‐masculine patients, the exogenous testosterone given as a part of some gender‐affirming care regimes may contribute to a progression of endometriotic lesions [52].

In our data, a more inclusive approach was desired by both gender‐diverse and cisgender respondents. There is some progress regarding the knowledge of endometriosis in the gender‐diverse AYA population, with the recent publication of a treatment guide for endometriosis in transmasculine adolescents [53]. Further research, both qualitative and quantitative, is needed to understand and improve the lived experience of gender‐diverse people with endometriosis.

## 5 Limitations and Strengths

This study was expected to capture some of the needs of endometriosis patients diverse in ethnicity, rurality, education level and disease severity, expanding and consolidating our previous findings [18, 29]. Notably, data collection occurred during the COVID‐19 pandemic and the subsequent lockdowns in Australia. This may have affected recruitment and the nature of participant responses [54]. This study reached a range of respondents in all demographic fields, although few respondents reported living in remote (1.5%) and very remote (2.3%) areas. As this survey was distributed on the internet, it is also likely to have captured a population of higher socioeconomic status (as an internet‐connected device is required) as well as a population experiencing higher anxiety levels relating to their disease [55]. Furthermore, as this survey was self‐selected and shared on support groups, it may represent those more severely affected by endometriosis and more dissatisfied with their care [56]. For these reasons, this study does not make claims as to the level of need experienced by this group or other groups but rather seeks to appreciate and report the range of needs experienced by AYAs with endometriosis and to understand some mechanisms underpinning those needs from the participants’ own perspectives.

One limitation of an anonymous online survey is the loss of the iterative process whereby researchers ask participants about the accuracy of the analysis. However, the responses in this data set were generally very clear and concise, and for the few responses that left leeway for interpretation, two researchers coded the response independently and discussed until a consensus on the content was reached. The sample size (*n* = 131), large for qualitative research, mitigated some of the interpretational uncertainty in the construction of themes and identifications of needs. Furthermore, the breadth of respondents and depth of some of the responses given facilitated a fruitful investigation.

## 6 Conclusion

Adolescents living with endometriosis are compelled to learn how to manage their education, work, health and relationships in parallel with their pain from a young age. As doctors begin to diagnose those with endometriosis earlier in life, this represents an opportunity to mitigate the detrimental impact that endometriosis can have on QoL. In identifying and exploring the supportive care needs of young people with endometriosis, we gain insight that may help facilitate improved shared decision‐making, appropriately allocate care and direct funding to maximise the QoL for these complex and often long‐standing patients.

## Author Contributions

**Louis Taffs:** Writing–original draft, methodology, writing–review & editing, investigation, formal analysis. **Niamh Waters:** Writing–original draft, methodology, formal analysis, writing–review & editing. **Jennifer Marino:** Investigation, writing–original draft, writing–review & editing, methodology, supervision, project administration. **Charlene Rapsey:** Conceptualisation, writing–original draft, writing–review & editing, supervision. **Michelle Peate:** Conceptualisation, investigation, funding acquisition, writing–original draft, writing–review & editing, methodology, supervision, project administration, resources. **Jane E. Girling:** Conceptualisation, investigation, writing–original draft, writing–review & editing, methodology, formal analysis, supervision, project administration.

## Ethics Statement

The design of the survey and the collection of the data were approved by the University of Melbourne Human Research and Ethics Committee (HREC) 2056522. All participants provided informed electronic consent.

## Conflicts of Interest

The authors declare no conflicts of interest.

## Data Availability

In accordance with the patient consent process of this study, the data from the open‐ended questions of this survey will not be made available. The anonymised demographic information is published in the accompanying tables.
